# Development of a High Performance Liquid Chromatographic Assay Measuring Mezlocillin in Serum or Tissue

**DOI:** 10.1155/S1064744993000171

**Published:** 1993

**Authors:** Sandra Mya, Ron E. Gain, Bryan Larsen

**Affiliations:** 1 Department of Obstetrics and Gynecology Marshall University School of Medicine Huntington WV USA; 2 Department of Biological Science Marshall University School of Medicine Huntington WV USA; 3 Department of Obstetrics and Gynecology Marshall University School of Medicine Huntington WV 25701 USA

## Abstract

*Objective:* This study evaluated the blood and uterine tissue concentration of mezlocillin, a broadspectrum penicillin.

*Methods:* We adapted a liquid chromatographic method to measure mezlocillin in serum and tissue. Mezlocillin reference standard was diluted in water, chromatographed on a reversed phase C18 column eluted at 1.5 ml/min with acetonitrile and phosphate buffer (1:3 v:v), and detected spectrophotometrically at 210 nm. Mezlocillin was administered to 14 premenopausal women scheduled to undergo vaginal hysterectomy. Each patient received a 4 g IV infusion of the drug 30 to 60 min prior to surgery. During surgery, tissue was removed from the uterine cervix and blood was obtained for assay of mezlocillin content.

*Results:* Chromatography of the mezlocillin standard furnished a discrete peak with a retention time of 2.4 min. The sensitivity of the assay was 0.1 µg/ml with a linear response up to 100 µg/ml. The correlation coefficient for the standard curve was 0.9997. When reference standard was diluted in pooled human serum, the assay was complicated by interfering compounds. These were removed by ether extraction. The sensitivity of the assay performed in serum was 3 µg/ml. Serum samples contained from 81.2 to 358 µg of mezlocillin/ml with an average serum concentration of 207.5 µg/ml. When serum containing a known amount of mezlocillin was homogenized for a period of time similar to that required to homogenize tissue samples, a deteatable loss of drug was observed and was applied as a correction factor to the measured tisulevels. After correction, the average tissue level was 117.2 µg/ml and ranged from 27% to 98% of the serum levels.

*Conclusions:* The serum concentration of mezlocillin after IV infusion of 4 g was greater than that required to inhibit the majority of the most significant organisms responsible for post-hysterectomy sepsis. Although tissue levels appeared to be consistently lower than serum levels, they could be expected to provide an inhibitory effect against many of the bacterial strains that contaminate the surgical site.


Infectious Diseases in Obstetrics and Gynecology 1:71–75 (1993)
© 1993 Wiley-Liss, Inc.

### Development of a High Performance Liquid Chromatographic Assay for Measuring Mezlocillin in Serum or Tissue

Sandra Mya, Ron E. Gain, and Bryan Larsen

*Departments of Obstetrics and Gynecology (S.M.), Biological Science (R.E.G.), and Microbiology and Obstetrics and Gynecology (B.L.), Marshall University School of Medicine, Huntington, WV*

### Abstract

***Objective:*** **This study evaluated the blood and uterine tissue concentration of mezlocillin, a broad-spectrum penicillin.**

***Methods:*** **We adapted a liquid chromatographic method to measure mezlocillin in serum and tissue. Mezlocillin reference standard was diluted in water, chromatographed on a reversed phase C18 column eluted at 1.5 ml/min with acetonitrile and phosphate buffer (1:3 v:v), and detected spectrophotometrically at 210 nm. Mezlocillin was administered to 14 premenopausal women scheduled to undergo vaginal hysterectomy. Each patient received a 4 g IV infusion of the drug 30 to 60 min prior to surgery. During surgery, tissue was removed from the uterine cervix and blood was obtained for assay of mezlocillin content.**

***Results:*** **Chromatography of the mezlocillin standard furnished a discrete peak with a retention time of 2.4 min. The sensitivity of the assay was 0.1 μg/ml with a linear response up to 100 μg/ml. The correlation coefficient for the standard curve was 0.9997. When reference standard was diluted in pooled human serum, the assay was complicated by interfering compounds. These were removed by ether extraction. The sensitivity of the assay performed in serum was 3 μg/ml. Serum samples contained from 81.2 to 358 μg of mezlocillin/ml with an average serum concentration of 207.5 μg/ml. When serum containing a known amount of mezlocillin was homogenized for a period of time similar to that required to homogenize tissue samples, a detectable loss of drug was observed and was applied as a correction factor to the measured tissue levels. After correction, the average tissue level was 117.2 μg/ml and ranged from 27% to 98% of the serum levels.**

***Conclusions:*** **The serum concentration of mezlocillin after IV infusion of 4 g was greater than that required to inhibit the majority of the most significant organisms responsible for post-hysterectomy sepsis. Although tissue levels appeared to be consistently lower than serum levels, they could be expected to provide an inhibitory effect against many of the bacterial strains that contaminate the surgical site.** © 1993 Wiley-Liss, Inc.

Key words
Antibiotics, pharmacodynamics, ureidopenicillin

Potent broad-spectrum congeners of penicillin are finding increasing use in a variety of clinical settings. The rational use of such antibacterial agents requires knowledge of the pharmacokinetics and pharmacodynamics of these drugs. Sensitive methods for assaying drugs in serum or tissue are needed for developing such information, and high performance liquid chromatography (HPLC) has become a useful technique for obtaining such information.

The present study adapted an HPLC method for analysis of serum and tissue samples from patients treated with mezlocillin. HPLC methods for de-

**Address correspondence/reprint requests to Dr. Bryan Larsen, Department of Microbiology, Marshall University School of Medicine, Huntington, WV 25701.**

Received December 23, 1992
Accepted August 12, 1993
Clinical Study termination of the related compounds mezlocillin and azlocillin in plasma have been reported,[1] but these methods have not been tailored for use with tissue. The methods available for measuring plasma levels were evaluated and adapted for use with serum and tissue specimens.

### MATERIALS AND METHODS

Mezlocillin as the sodium salt with a potency of 920 μg/mg was provided by Miles Pharmaceuticals (New Haven, CT). The drug was reconstituted with distilled water to make a stock solution of 100.28 μg/ml. Stock solutions were stored at −74°C.

The 14 patients studied were scheduled to undergo vaginal hysterectomy and were given preoperative prophylaxis consisting of 4 g mezlocillin dissolved in 500 ml of normal saline by IV infusion. The antibiotic infusion was started within 1 hr of the time the surgical procedure began.

During the surgical procedure, when the uterus was being removed, a blood sample was drawn from the antecubital vein, and the blood was allowed to clot. A section was cut from the uterine cervix, and both blood and tissue were transported to the laboratory, where the serum was removed from the clot and serum and tissue stored at −74°C until analysis.

The method for tissue and serum analysis was established experimentally (see Results). Prior to assay, tissue and blood samples were thawed at room temperature. Approximately 0.25 g was cut from the tissue sample, weighed to the nearest 0.1 mg, minced, and placed in a tube containing 3 ml of water. These samples were kept on ice until the tissue was liquified with a Tekmar tissue homogenizer (Tekmar, Cincinnati, OH). The homogenate was deproteinized by the addition of 5 ml acetonitrile, followed by centrifugation for 15 min at 2,000*g*. The supernatant fluid was removed and extracted 3 times with 3 ml of diethyl ether. The aqueous phase was then injected onto the chromatograph.

The serum samples were deproteinized with equal volumes of acetonitrile, centrifuged at 2,000*g* for 15 min, and extracted 3 times with 2.5 volumes of diethyl ether. The aqueous phase was chromatographed.

Samples were analyzed by HPLC on a 4.6 × 220 mm octadecyl silyl, 5 μm particle size column with a guard column of identical composition. Samples of 20 μl were injected and eluted with 1:3 acetonitrile:0.01 M potassium dihydrogen phosphate buffer, pH 7, at a flow rate of 1.5 ml/min. The column effluent was monitored spectrophotometrically at 210 nm. Except where specifically noted, all measurements were made by triplicate determinations on each sample, and the identity of mezlocillin was based on retention time while quantitation was based on peak height.

Dilutions of mezlocillin standard were made in water or pooled human serum to determine linearity, repeatability, and sensitivity of the assays. Standard curves made in serum were used to establish drug levels in the patient specimens.

A control to determine whether drug may have been lost during the process of tissue disruption was prepared. Serum containing a known amount of mezlocillin was homogenized for a period of time equal to the time required to disrupt the tissue samples. This control was then subjected to deproteinization and ether extraction as for other samples. The peak height obtained from this sample was compared with the result obtained from a serum sample with an identical concentration of mezlocillin that had not undergone homogenization. This provided a correction factor that was applied to tissue concentrations to correct for drug loss due to tissue homogenization.

### RESULTS

Figure 1 shows that the mezlocillin standard diluted in water produced a symmetrical peak with a retention time of 2.4 min. Thus, the chromatographic method used was deemed acceptable for detecting the pure compound.

A series of dilutions of mezlocillin standard from 0.1 μg/ml to 100.28 μg/ml were made in water to allow determination of the sensitivity and linearity of the assay method. The smallest concentration detected was 0.1 μg/ml, which furnished an average peak height of 0.00083 absorbance units at 210 nm. The assay was linear over the full range of concentrations and provided a correlation coefficient ($r^2$) of 0.9997.

Ten replicate chromatograms of a standard containing 100.28 μg mezlocillin standard/ml were evaluated to determine the repeatability of peak

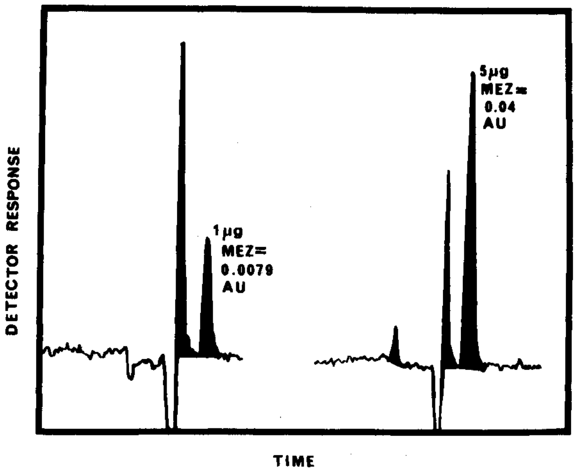

**Fig. 1. Mezlocillin (MEZ) reference standard diluted in water chromatographed on a reversed phase column under conditions described in the Materials and Methods section. A single symmetrical peak representing the pure compound was observed. Chromatography of a larger concentration revealed a linear relationship between peak height and concentration.**

height and retention time. The average retention time was 2.40 ± 0.024 min (SD). The average peak height was 0.774 ± 0.0118 absorbance units (SD). The coefficient of variation (relative SD) for retention time was 1% and for peak height was 1.5%. The procedure was therefore considered to be highly repeatable.

The methods for preparation of serum samples for chromatographic determination of mezlocillin were evaluated. A known concentration of mezlocillin was added to a pool of human serum, and various methods were used to prepare aliquots of that serum for analysis. The first method involved addition of 1 ml of 5% perchloric acid to 1 ml of the serum sample followed by centrifugation. This provided a protein-free sample for chromatography. As illustrated in Figure 2, this method of preparation was unsuitable because exposure to perchloric acid appeared to generate at least two new peaks, which may have been degradation products of mezlocillin. Furthermore, it was not possible to identify the mezlocillin peak unequivocally in the acid-treated serum.

The second method of sample preparation employed the addition of 1 ml of acetonitrile to 1 ml of serum to precipitate the protein from the sample. It was believed that the acetonitrile would be less likely to cause degradation of the mezlocillin than would perchloric acid. However, the protein-free supernatant fluid from acetonitrile-treated serum yielded a chromatogram similar to that obtained when serum without mezlocillin was treated with acetonitrile. This finding suggested that the mezlocillin was being masked by some component of the serum.

It was subsequently found that diethyl ether extraction removed the interfering compounds from the acetonitrile-precipitated samples, as shown in Figure 3. In addition, the acetonitrile entered the ether phase, which eliminated the dilution of the sample due to the deproteinization step. Consequently, the acetonitrile precipitation followed by ether extraction was adopted for sample preparation.

When standards prepared in water, treated with acetonitrile, and extracted with ether were compared with standards diluted in water, no appreciable loss of drug due to the extraction procedure was observed. Recoveries ranged from 97.7% to 106% for the concentrations of mezlocillin observed in the patient samples. Based on these findings, standard curves for mezlocillin assay were prepared in pooled serum, and patient samples were compared with these standards.

Tissue samples were also compared with a standard curve prepared in serum. However, it was determined that the tissue homogenization was expected to have reduced the amount of mezlocillin detectable by 37.5% based on homogenizing serum samples with added mezlocillin. As a consequence of this finding, the observed tissue values were increased by a factor of 0.375.

The serum and tissue levels observed in the 14 patients studied are summarized in Table 1. The levels observed in serum were consistently higher than those found in tissue. The highest serum levels tended to be associated with the highest tissue levels, but the relationship of serum to tissue levels revealed an $r^2$ of 0.58.

This study was not designed to be a pharmacokinetic study, but rather an evaluation of the serum and tissue levels obtained simultaneously after IV infusion. Therefore, the time after infusion that the tissue sample was removed and the serum sample was taken was not identical in each case and varied from 5 min to 130 min. In the limited number of samples available in the study, no linear correlation between the time after infusion and tissue or serum levels was apparent.

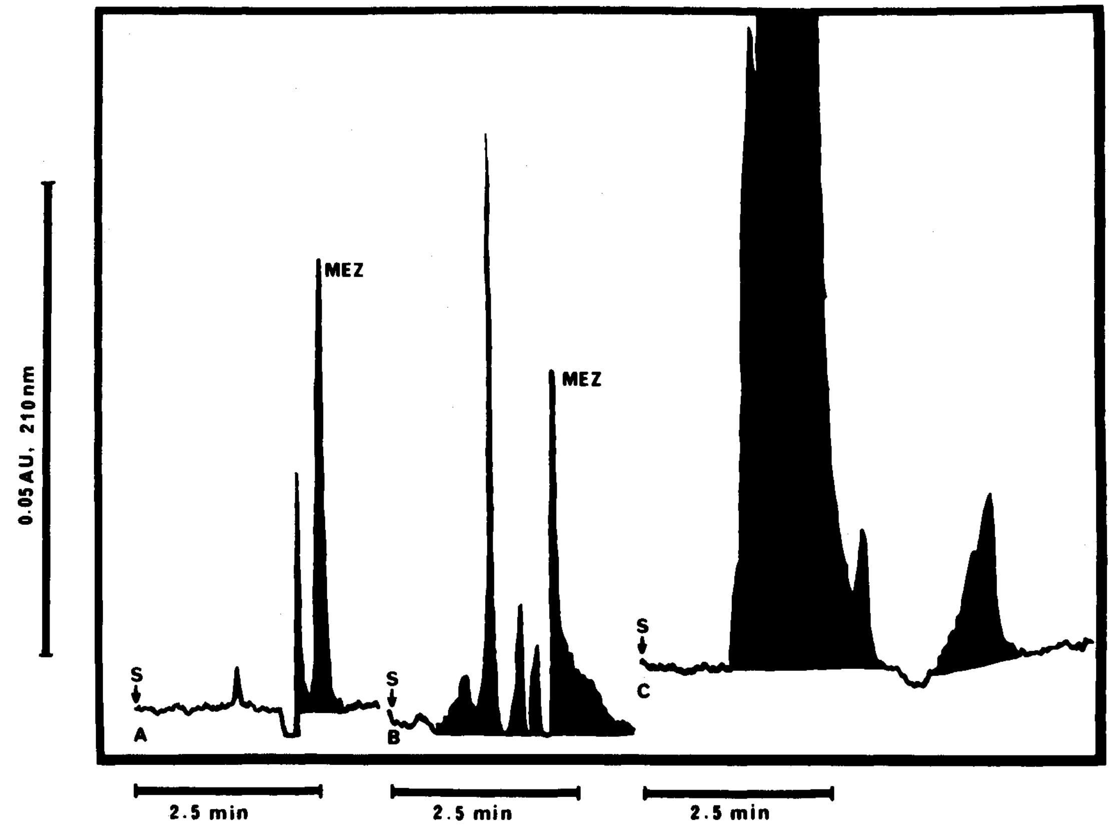

Fig. 2. Evaluation by chromatograms of perchloric acid precipitation for preparation of serum samples. **A:** Mezlocillin (MEZ) standard (6.25 μg/ml, final concentration) diluted in water with a retention time of approximately 2.4 min. **B:** Mezlocillin, 6.25 μg/ml, with 2.5% perchloric acid. The height of the mezlocillin peak was diminished (compared with A), and multiple new peaks appeared, suggesting degradation products. **C:** Mezlocillin, 6.25 μg/ml (final concentration), in serum deproteinized with perchloric acid, showing that mezlocilin is obscured by serum components. S: Start of each chromatographic run.

### DISCUSSION

The use of antimicrobial prophylaxis in such surgical procedures as vaginal or abdominal hysterectomy is so common that one may be tempted to consider studies involving prophylaxis as trivial. Despite the frequent application of prophylaxis, the fundamental reasons why this type of drug use is effective is still a matter of conjecture. It is assumed that antibiotics administered preoperatively affect microorganisms that contaminate the operative site, but it is not clear whether any of the organisms are killed, prevented from replicating, or simply prevented from producing virulence factors. It is likewise not clear what relevance in vitro minimal inhibitory concentrations have for these tissues. The length of time and concentration of drug required to be present in the tissue for effective prophylaxis

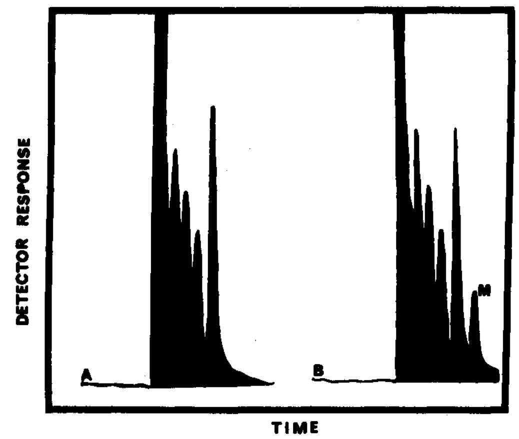

Fig. 3. **A:** Pattern obtained when ether-extracted serum is chromatographed. **B:** Results when the serum is spiked with mezlocillin (M) standard and extracted with ether. The mezlocillin produces a resolved and quantifiable peak.

**TABLE 1. Observed concentrations of mezlocillin in serum and uterine cervix from women given 4 g of mezlocillin prior to vaginal hysterectomy**

| | Concentration (μg/g) | | Ratio |
|---|---|---|---|
| | Tissue | Serum | (tissue/serum) |
| Mean | 117.2 | 207.5 | 0.567 |
| Standard deviation | 66.9 | 93.0 | 0.233 |
| Range | 27.5–228.1 | 81.2–358.0 | 0.268–0.982 |

are also open to question. Analysis of tissue levels of various antimicrobials as well as serum levels represents a necessary tool for enhancing our understanding of the effects of prophylaxis at the operative site.

The work presented here adapted a chromatographic procedure for use in determining the serum and tissue levels of mezlocillin. An attempt was made to take into account the possibility that interaction of the drug with tissue or serum components may reduce the amount of drug apparent in our assays. It cannot be claimed that subjecting mezlocillin-spiked serum to the homogenization step provided a true control for possible loss in tissue samples, although the data suggest that such an observation is not irrelevant. An ideal study would have added drug to tissue from a patient not treated with antibiotic and processed the tissue as described; however, obtaining tissue samples from patients who had not received the drug was outside the bounds of the protocol approved by the IRB, leaving the serum homogenization test as the suboptimal alternative. Certainly, any future application of this method could be improved by including a control using tissue from a patient not exposed to antibiotic.

The question of why the homogenizing procedure made a portion of the drug unrecoverable is not explained by the studies presented. Oxidative damage to the drug or some irreversible reaction with protein or other constituents of the biological matrix are potential explanations. The possibility that drug was incorporated into micelles during the homogenization process may be less likely, since the solvent extraction steps should destroy such structures.

In view of the above considerations, it may be stated that in all cases, some mezlocillin was present in uterine tissue at the time of incision, and the tissue concentration was at least one quarter that present in serum if one accepts the correction factor. A more conservative estimate (without the correction factor) of tissue concentrations ranges from 17.2 to 142.5 μg/g and would average 35% of the simultaneous tissue values.

For therapy of established infections, it is desirable to achieve serum and tissue concentrations greater (generally by 2–4 times) than the minimal inhibitory concentrations of the etiologic agents. Somewhat less certain are the serum or tissue levels required to achieve prophylaxis against infection of the operative site. It is not clear whether it is necessary to achieve minimal inhibitory concentrations against microorganisms at the surgical site. The levels of mezlocillin observed in serum and tissue are well above the minimal inhibitory concentrations reported for some of the microorganisms commonly involved in infections after hysterectomy.[2] For example, 90% of *Bacteroides fragilis* strains are inhibited by 32 μg of mezlocillin/ml and 90% of *Escherichia coli* strains are inhibited by 64 μg/ml. It should be noted, however, that minimal inhibitory concentrations are determined in vitro with a constant concentration of drug and with the test organism exposed to that drug for many hours. In the patient, the concentration of the drug is constantly changing. The present study, not being a pharmacokinetic study, did not sample tissue and serum at identical times after infusion. Thus, while significant levels of antibiotic were detected in the patients studied, future work will need to elucidate the relevance of brief exposures of microorganisms to these concentrations of mezlocillin. This chromatographic technique has proved an appropriate tool in establishing the concentration of mezlocillin to be studied in future investigations.

### REFERENCES

1. Rouan MC: Antibiotic monitoring in body fluids. J Chromatogr 340:361–400, 1985.
2. Parry MF, Pancoast SJ: Antipseudomonal penicillins. In Cunha B, Ristuccia A (eds): Antimicrobial Therapy. New York: Raven Press, pp 197–208, 1984.
